# Supplementary figures and images for: Analytical code sharing practices in biomedical research
Source: PeerJ Comput Sci. 2024 Jun 28;10:e2066. doi: 10.7717/peerj-cs.2066 (PMC11232620; doi:10.7717/peerj-cs.2066)

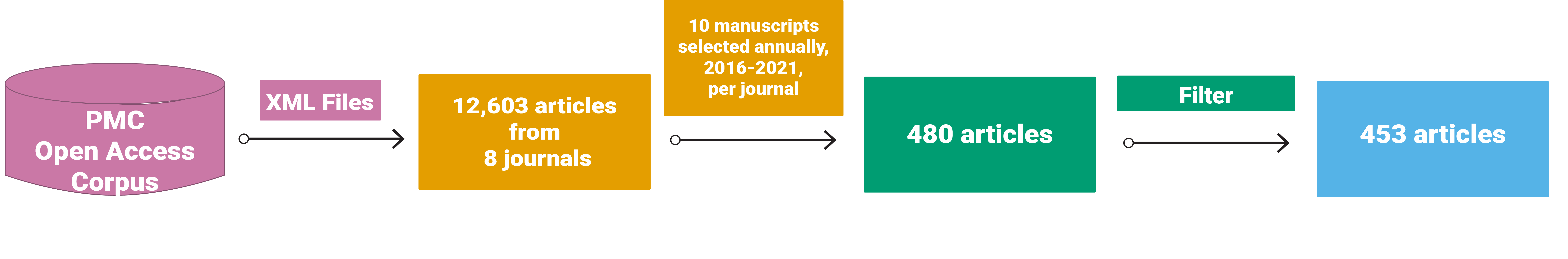

Supplement: Supplemental Information 1 — Each shape represents a distinct segment of the workflow, spanning from article retrieval to the selection of articles utilized for analysis. [file peerj-cs-10-2066-s001.png]

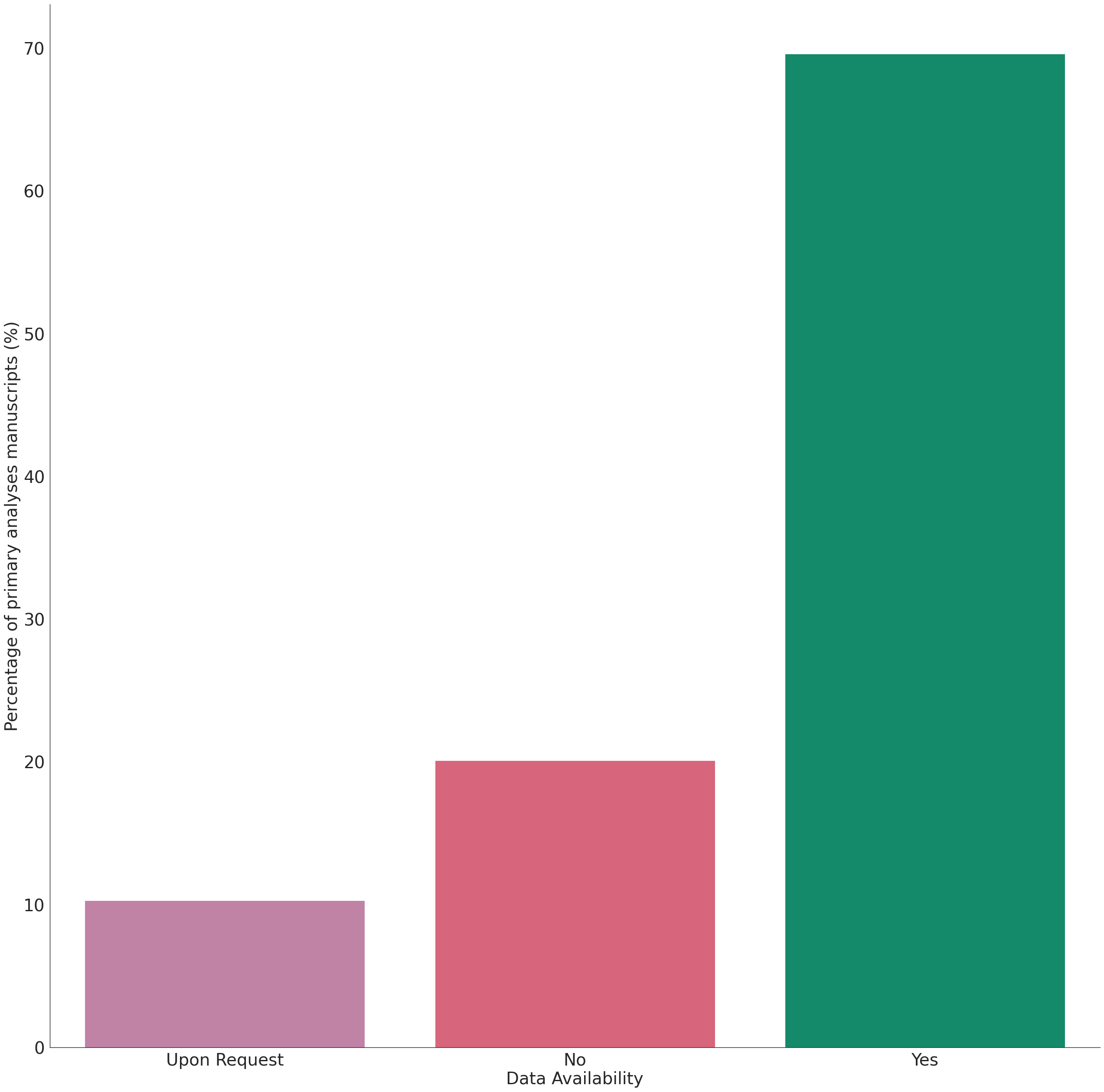

Supplement: Supplemental Information 2 — Each bar illustrates the percentage of primary analysis studies with available data, categorized into the following groups: "Upon Request," "No," and "Yes", (n=204). [file peerj-cs-10-2066-s002.png]

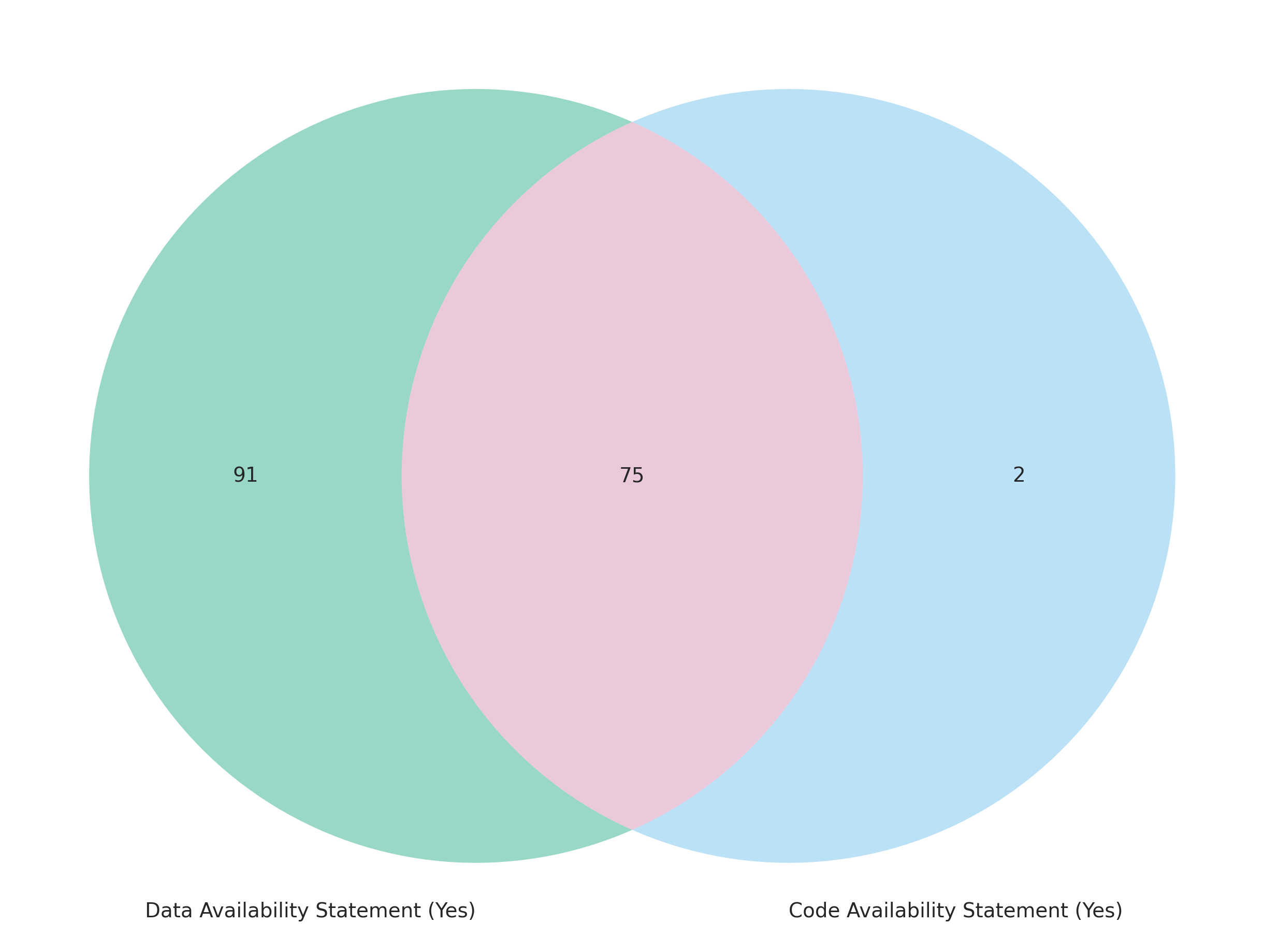

Supplement: Supplemental Information 3 — Each number enclosed within a circle denotes the count of primary analysis manuscripts categorized into the following groups: those with solely a data availability statement, those with both data and code availability statements, and those with solely a code availability statement, (n=204). [file peerj-cs-10-2066-s003.png]

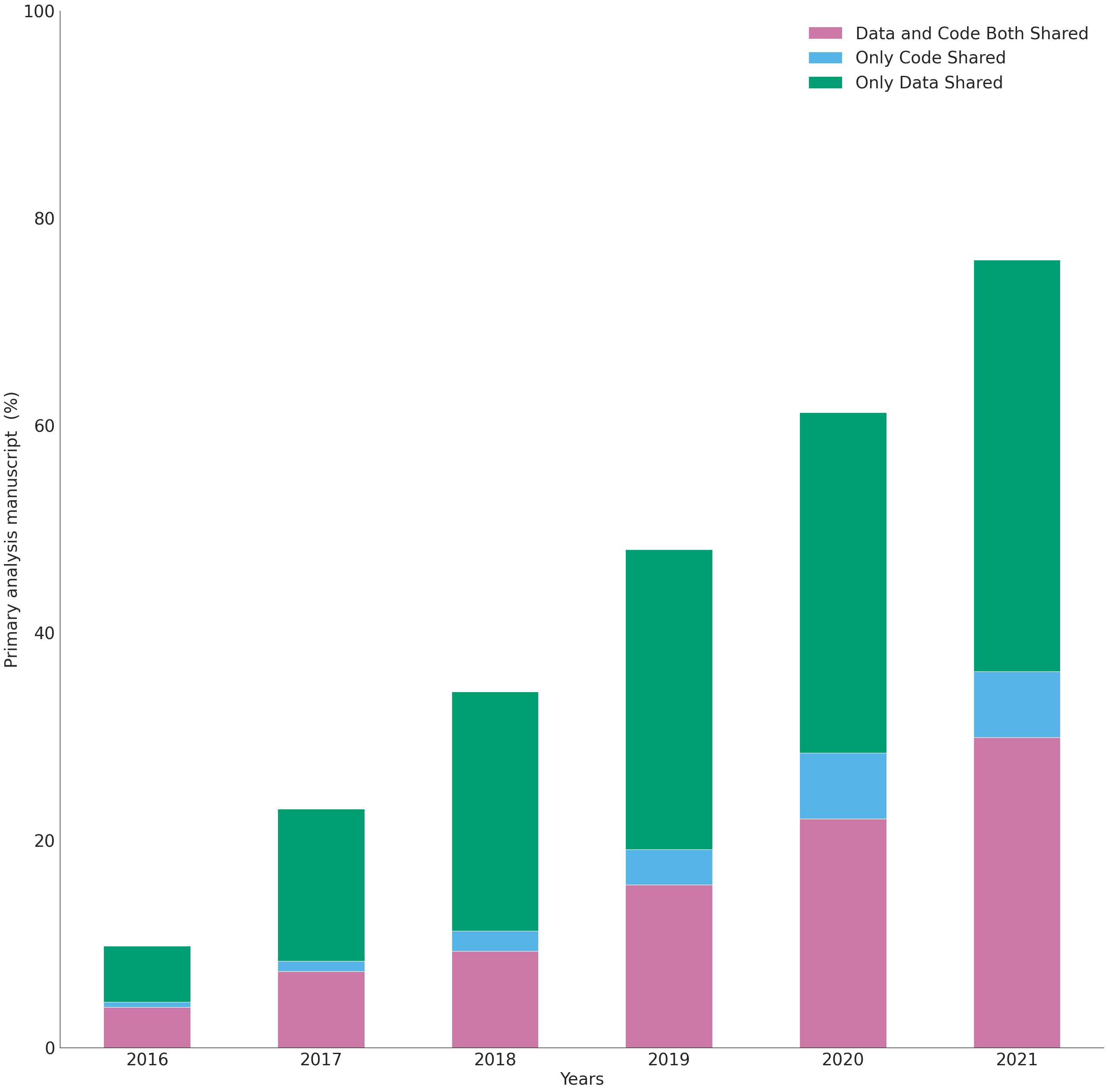

Supplement: Supplemental Information 4 — A cumulative plot illustrating data and code sharing for primary analysis manuscripts from 2016 to 2021. Each bar is stacked, representing the proportion of primary analysis articles over the years, depicting whether the article shared only data, only code, or both. The bars are plotted year by year to showcase the cumulative growth over time, (n=204). [file peerj-cs-10-2066-s004.png]

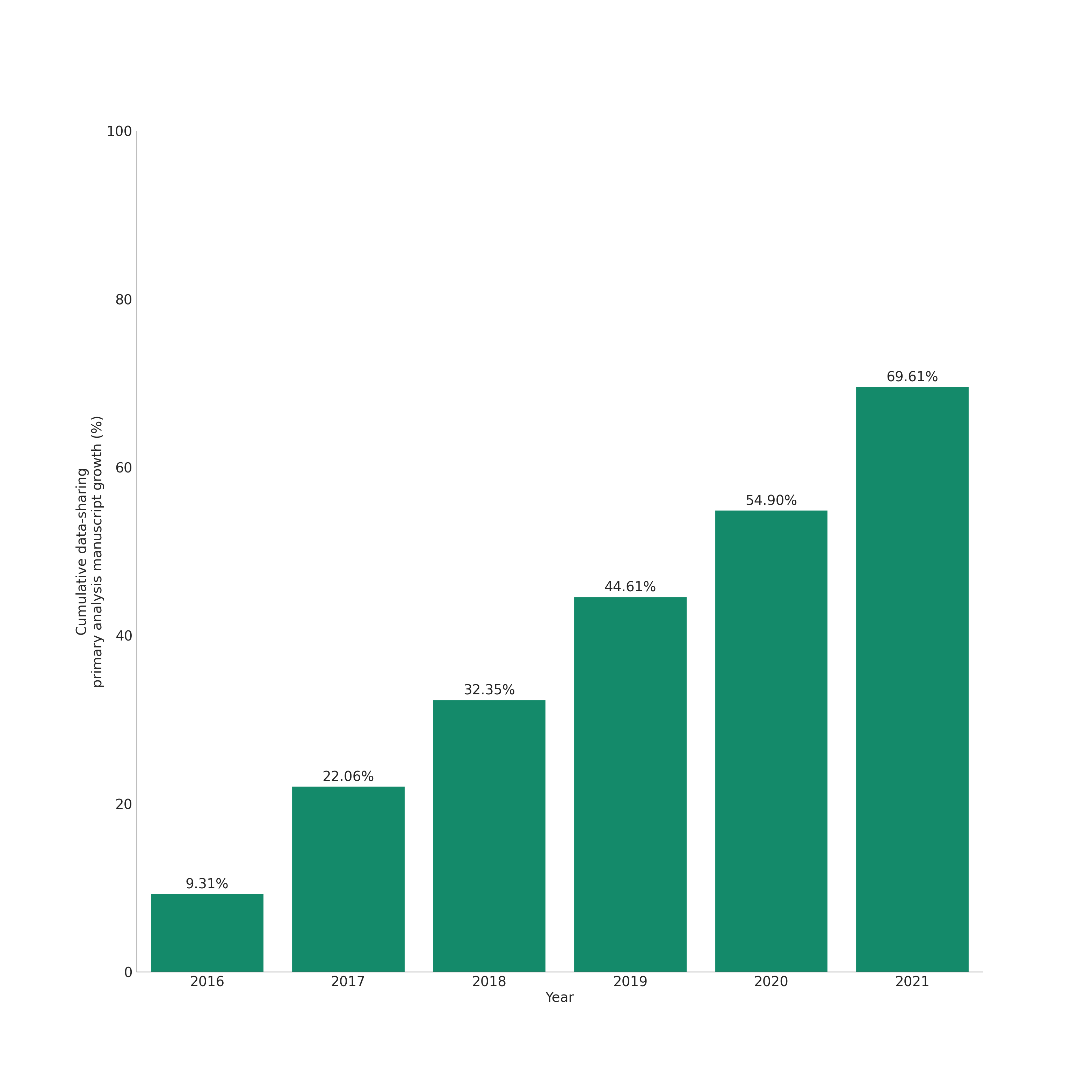

Supplement: Supplemental Information 5 — Each bar depicts the cumulative percentage of data sharing in primary analysis manuscripts per year, (n=204). [file peerj-cs-10-2066-s005.png]

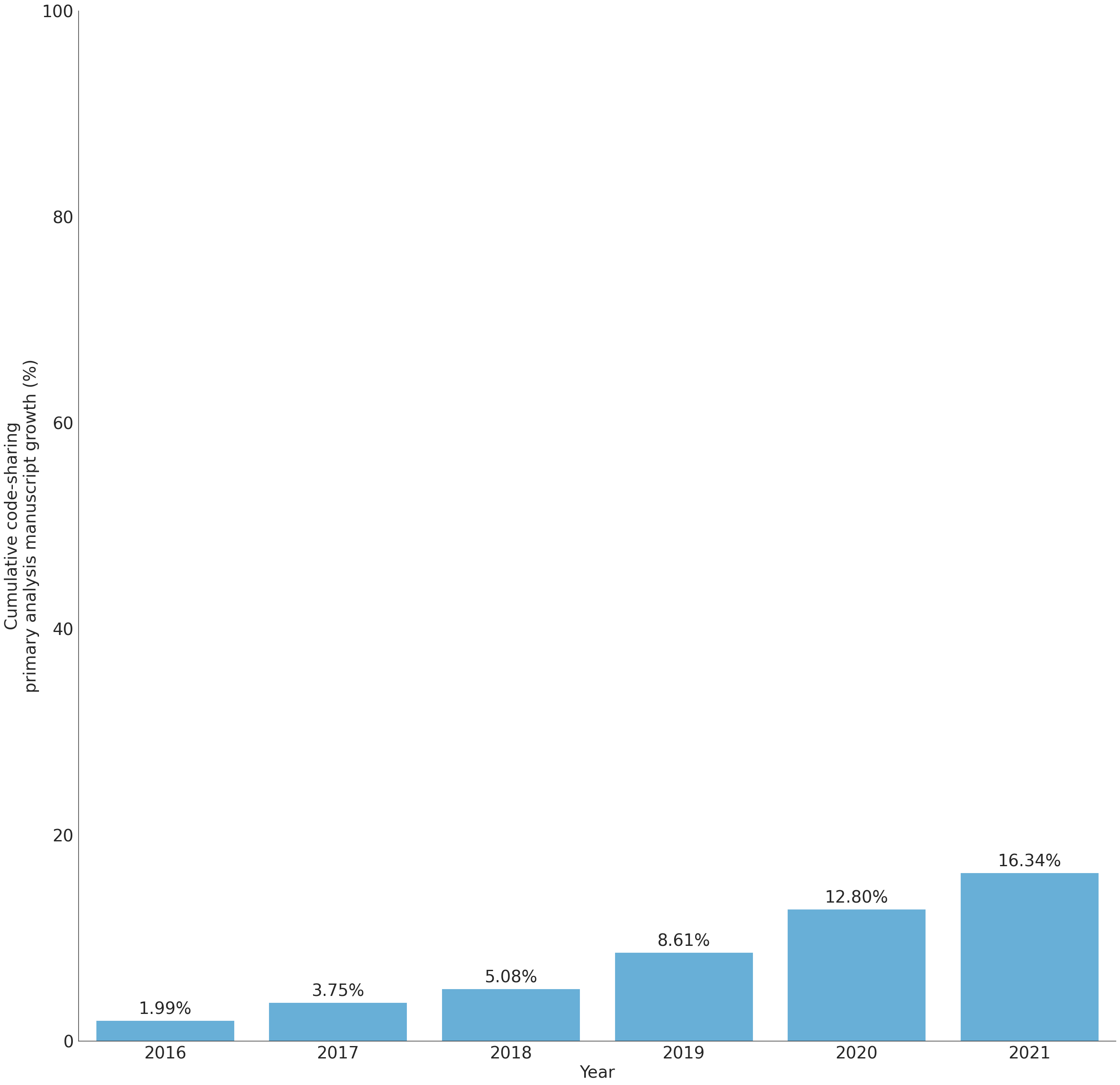

Supplement: Supplemental Information 6 — Each bar illustrates the cumulative growth of code sharing in primary analysis manuscripts per year, (n=204). [file peerj-cs-10-2066-s006.png]

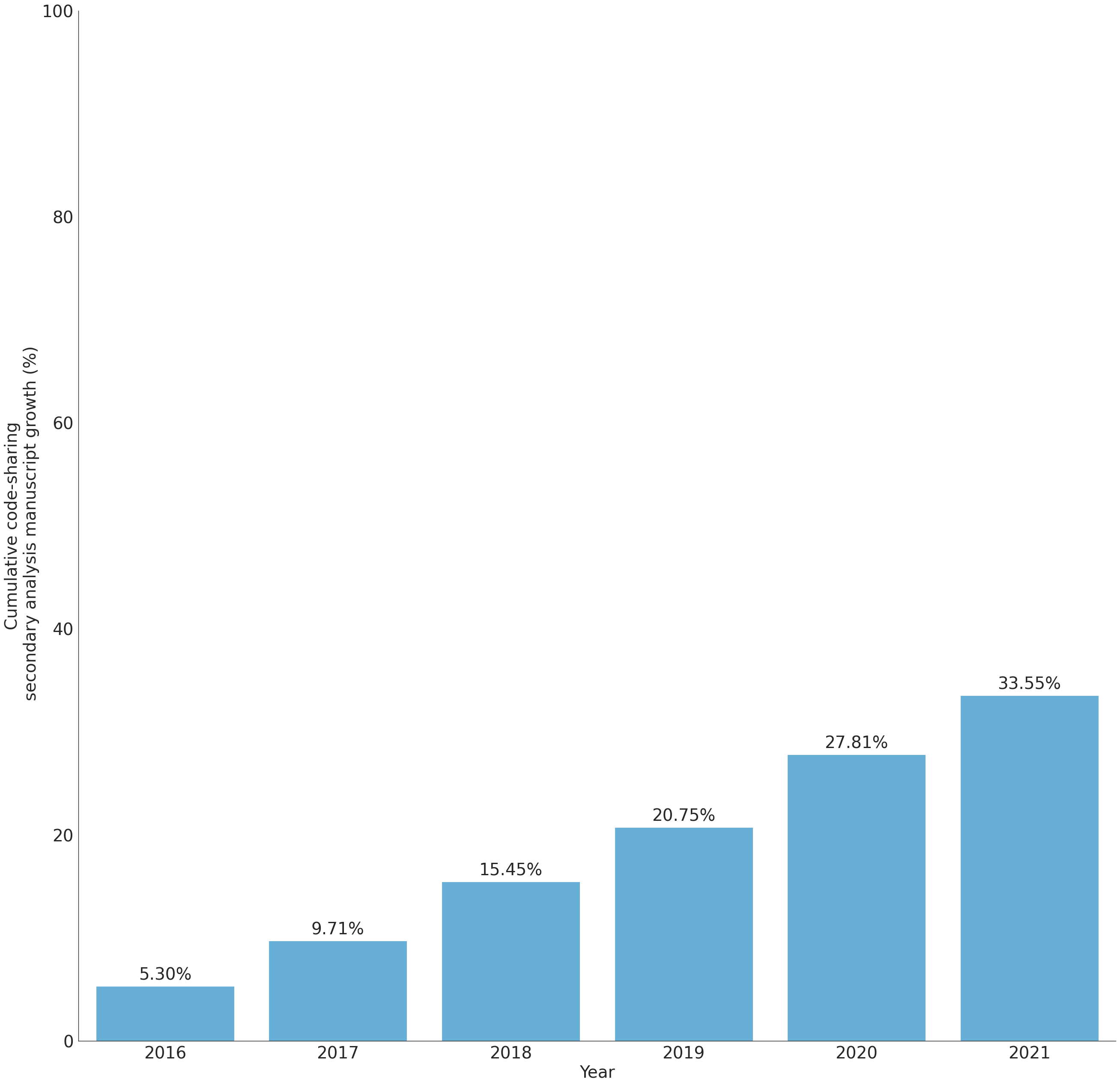

Supplement: Supplemental Information 7 — Each bar represents the cumulative growth of code sharing in secondary analysis manuscripts by year, (n=249). [file peerj-cs-10-2066-s007.png]

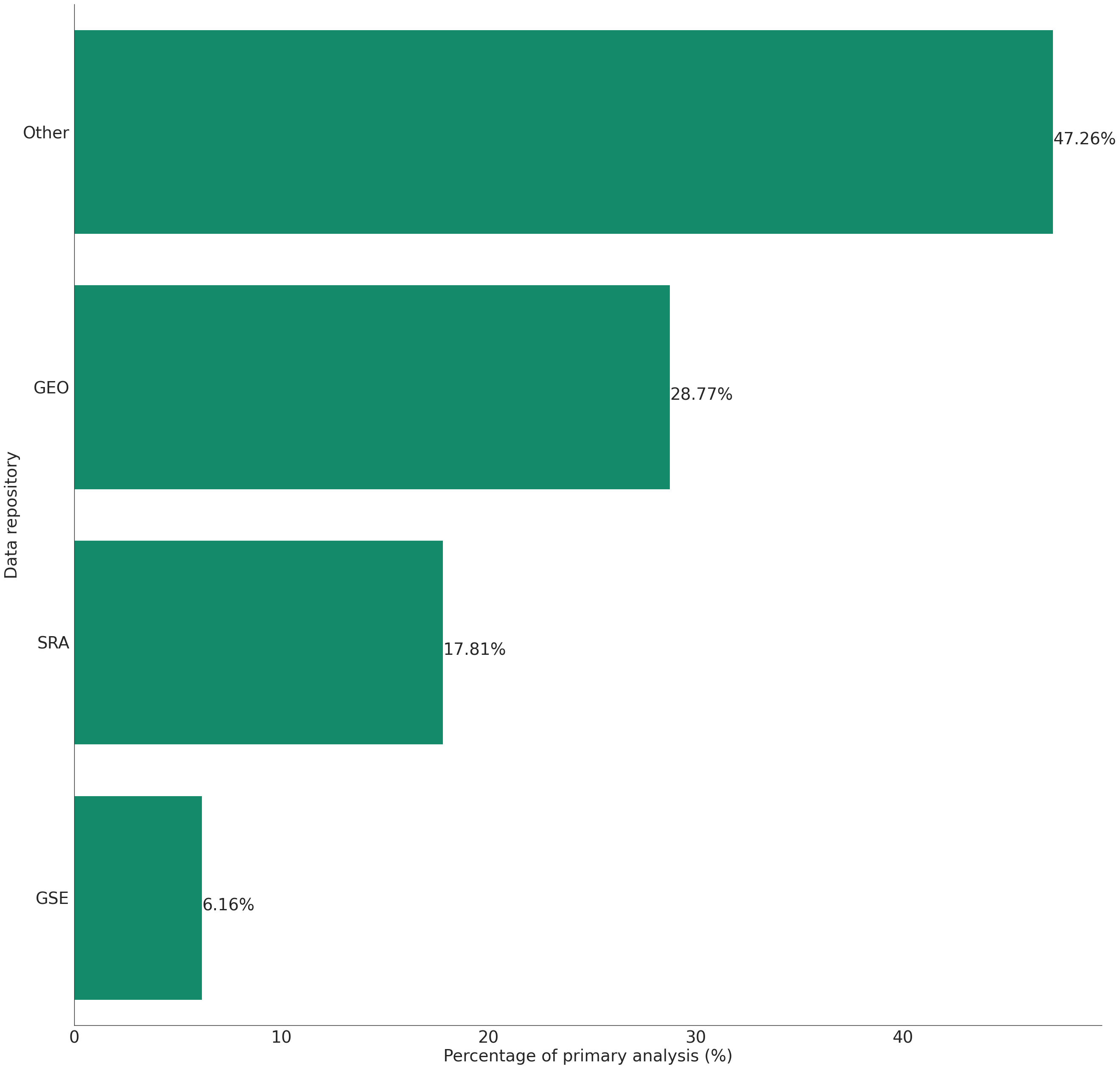

Supplement: Supplemental Information 8 — Each bar represents the percentage of primary analysis articles utilizing one of the following repositories for data storage: GEO (Gene Expression Omnibus), SRA (Sequence Read Archive), and GSE (Gene Expression Omnibus). "Other" encompasses a compilation of repositories that were less frequently utilized, facilitating a concise depiction, (n=204). [file peerj-cs-10-2066-s008.png]

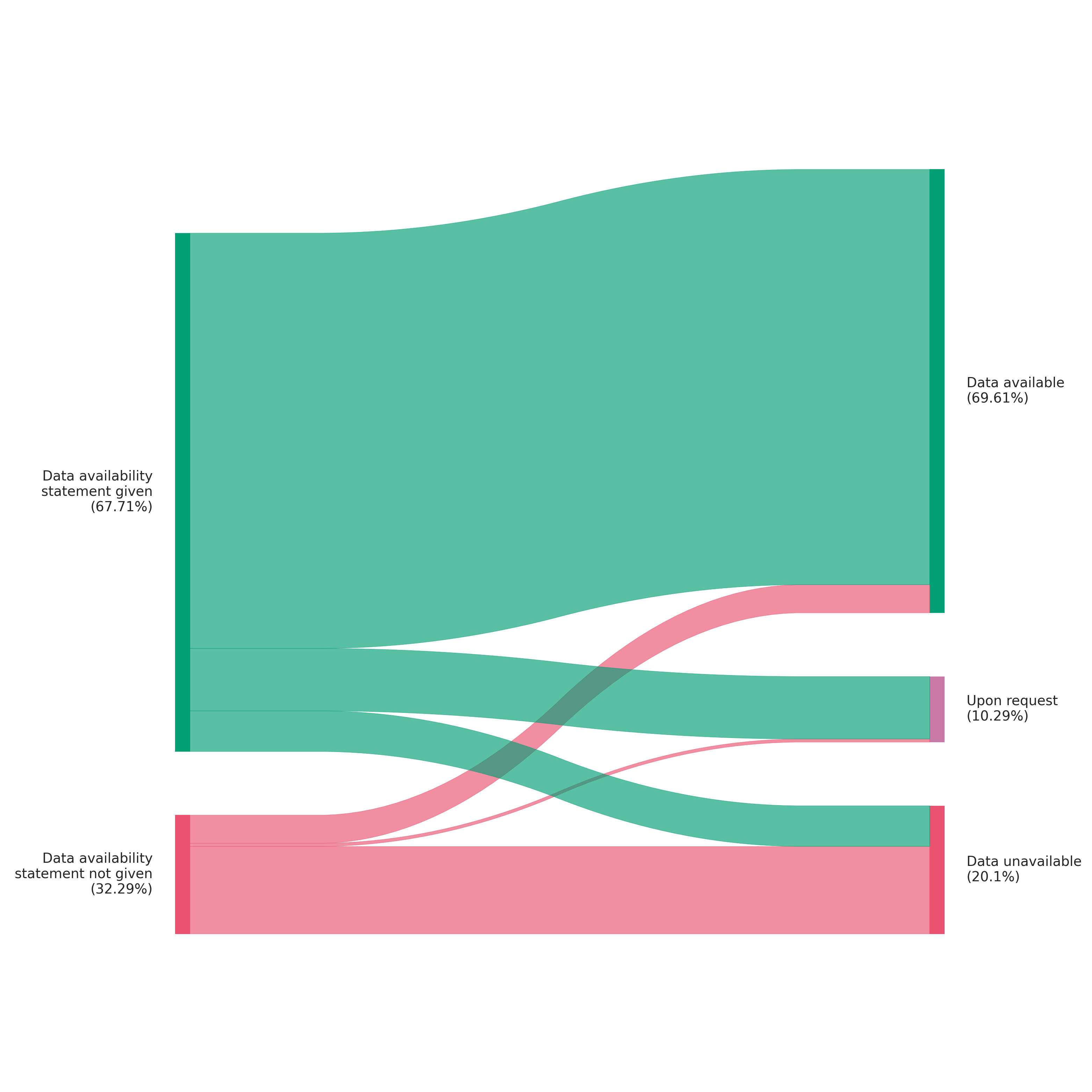

Supplement: Supplemental Information 9 — This Sankey diagram illustrates the flow of manuscripts among five categories: Manuscripts with a Data Availability Statement Given and Data Availability Statement Not Given Transitioning to Data Being Actually Available, Data Available Upon Request. Data Being Unavailable: Under each label is the percentage of articles that fit into that category (n=453). [file peerj-cs-10-2066-s009.png]

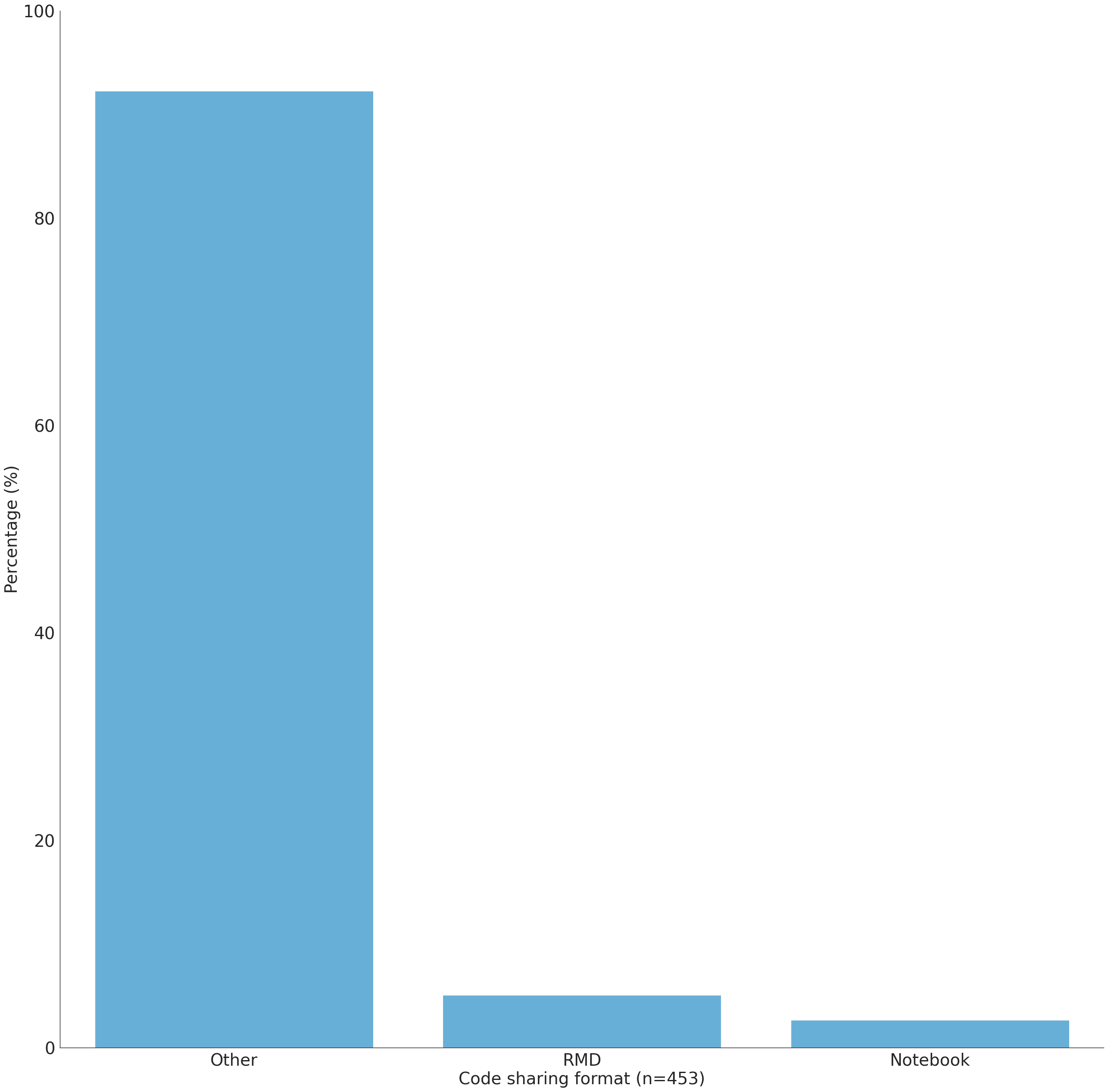

Supplement: Supplemental Information 10 — Each bar represents the percentage of all articles sharing their code across various formats: RMD (R Markdown Document), Notebook (Jupyter Notebook or other notebook-based formats), and Other, which includes diverse formats like plain scripts, among others (n=453). [file peerj-cs-10-2066-s010.png]

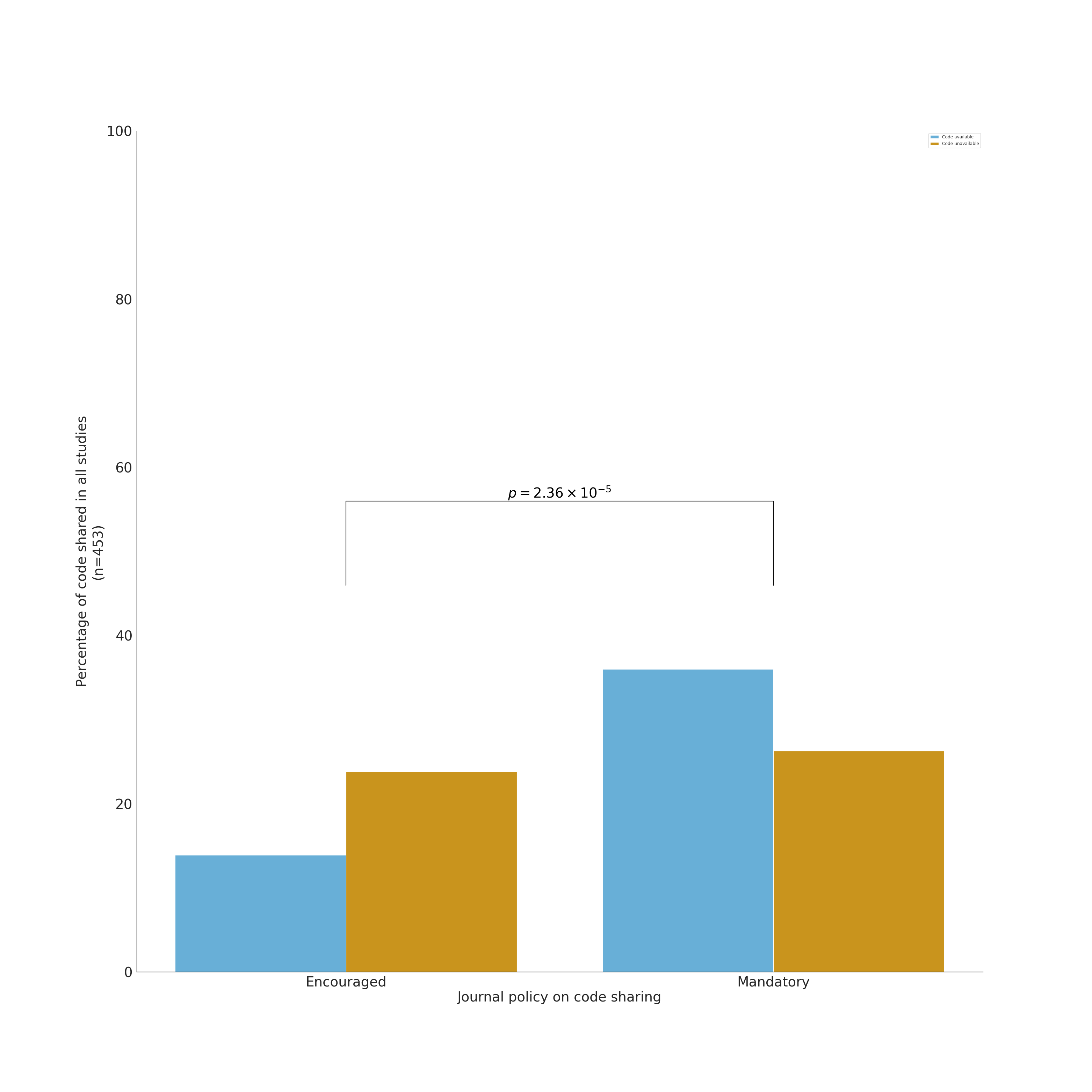

Supplement: Supplemental Information 11 — Each bar illustrates the percentage of articles sharing code based on journal policy: Mandatory or Encouraged. Articles submitted to journals with a Mandatory policy were obligated to share code, while those submitted to journals with an Encouraged policy were merely encouraged to share code, without being mandatory (n=453). [file peerj-cs-10-2066-s011.png]
