# Supplementary material for: Analytical code sharing practices in biomedical research
Source: PeerJ Comput Sci. 2024 Jun 28;10:e2066. doi: 10.7717/peerj-cs.2066 (PMC11232620; doi:10.7717/peerj-cs.2066)
Supplement: Supplemental Information 12 — Each row represents the source/platform utilized by the articles to store their code, displaying both the raw counts of articles opting for each platform and the percentage of articles selecting each platform (n=453). [file peerj-cs-10-2066-s012.docx]

**Supplementary Table 1**: Availability of code across 453 biomedical articles classified according to the code repository.

| **Source** | **Counts** | **Percentage** |
| --- | --- | --- |
| 10xGenomics | 1 | 0.43 |
| Downloadable File | 2 | 0.85 |
| GitLab | 2 | 0.85 |
| CRAN | 2 | 0.85 |
| Bioconductor | 2 | 0.85 |
| Sourceforge | 3 | 1.28 |
| Bitbucket | 6 | 2.56 |
| Supplementary | 6 | 2.56 |
| Zenodo | 14 | 5.98 |
| Website | 16 | 6.84 |
| GitHub | 180 | 76.92 |
